# Supplementary material for: NF2 Alteration/22q Loss Is Associated with Recurrence in WHO Grade 1 Sphenoid Wing Meningiomas
Source: Cancers (Basel). 2022 Jun 29;14(13):3183. doi: 10.3390/cancers14133183 (PMC9265038; doi:10.3390/cancers14133183)
Supplement: Supplementary file 1 [file cancers-14-03183-s001.zip › cancers-1733968 supplementary material updated.pdf]

cancers

MDPI

# Supplementary Material: *NF2* Alteration/22q Loss Is Associated with Recurrence in WHO Grade 1 Sphenoid Wing Meningiomas

**Yu Sakai, Satoru Miyawaki*, Yu Teranishi, Atsushi Okano, Kenta Ohara, Hiroki Hongo, Daiichiro Ishigami, Daisuke Shimada, Jun Mitsui, Hirofumi Nakatomi and Nobuhito Saito**

**Table S1.** Primer information.

| Gene | exon | Forward primer | Reverse primer |
|---|---|---|---|
| *NF2* | 1 | GGGCTAAAGGGCTCAGAGTG | ACCTCTCGAGCTTCCAC-CTC |
| | 2 | AGTGTCATCCCCAC-GTTTTG | CCCCAG-TGATGAGCTAGGC |
| | 3 | TTGCAAAGGCTTCTTT-GAGG | AACTCTGCAACCAC-TCCTGG |
| | 4 | GCCATCTGTT-GTGATCAGCC | TCCCATGACCCAAATTAACG |
| | 5 | TGTTCAGAAATGGCAG-TTATCTTTAG | CCTTCAAGTCCTTT-GGTTAGC |
| | 6 | CTCTGTGTGAC-TATCTCCCTGG | CAAGCATGTCCTAGTTTT-GCAG |
| | 7 | AATGCTTGATTT-GGTGCCC | AGTCTGGCCCTCAC-TCAGTC |
| | 8 | GTGCCAGATTCTTT-GGAAGG | GGGGCAGACAGGGAAAG |
| | 9 | CCAATTGCTGG-TAACATTCC | CATTCTATACTTCACAA-GATGTCACTC |
| | 10 | TAGTGGGCCAG-TAGGCAGTG | GGCCAGGACTGAC-CACAC |
| | 11 | CTTGTGG-CACCCTAGGTCTC | TCAAAGCAAAC-GCTGCTAAC |
| | 12 | CCCATCTCAG-TGTTCAAGGC | CTGGTCTGCGGCCCTTC |
| | 13 | TGTCCTTTTTCAC-CTCTTTGG | GTGTTT-GCCTGAATGGTCAC |
| | 14 | AGGATCGGTT-GTCAACACAG | AGGCCCCAATCAC-TCAGTC |
| | 15 | GAGCCGTGTCTCAC-TGTCTG | AGGAAAC-CAGATGCCAACC |
| | 16 | AGGACAG-GACCCTGTGTGAC | TTGA-TATCTGGTCCATCCCG |
| *TRAF7* | 12/13 | ATCAGGGGTCTT-GTGTGTGG | CAGGAATGAG-TGAGGGAGCC |
| | 14 | TATGGGTGGGAC-CTTCTGGG | CCCTATAGGTGGGG-CACACA |
| | 15/16/17 | CTGATGGCTGG-CATGGAC | GAAGGTAGGAACAGGG-CAGA |
| | 18/19 | CTGCCCTGTTCCTAC-CTTCG | GTGACAC-TGCCCTGGTGAC |
| | 20/21 | CGCCAGACCAGAC-CAAAGTC | CAGAGCCTGTCCAC-CTATGC |

| | | | |
|---|---|---|---|
| *AKT1* | 2 | GGTAGAG-TGTGCGTGGCTCT | CGCCACAGAGAAGTT-GTTGA |
| *KLF4* | 3 | TTCACCCCTA-GAGCTCATGC | TCTGCAGTTT-GTCCCCCTAC |
| *SMO* | 7 | TTCACCCCTA-GAGCTCATGC | TCTGCAGTTT-GTCCCCCTAC |
| | 10 | CCCATCCCTGACTGTGA-GAT | TGACCAAGGCTGTGCTA-GAG |
| *POLR2A* | 8 | TCGAGAG-TCTGCTCCTCCTA | AGCCTGCCCTATCTT-GGAAAG |
| *PIK3CA* | 9 | ATCATCTGTGAATCCAGA | TTAGCACTTACCTGTGAC |
| | 20 | CTCTGGAATGCCAGAAC-TAC | ATGCTGTTTAATTGTGTG-GAAG |
| *TERT* | | CCAGCTCCGCCTCCTCCG | GCTGCCTGAAACTCGCGCC |

**Table S2.** Composition of PCR reaction mixtures.

| Reagent | Concentration | Volume(µl) |
|---|---|---|
| Double Distilled Water | | 3.5 |
| dNTP Mix | 2mM | 4 |
| Buffer (2x) | | 10 |
| Forward primer | 20µM | 0.5 |
| Reverse primer | 20µM | 0.5 |
| DNA polymerase | | 0.5 |
| DNA template | | 1 |
| Total | | 20 |

**Table S3.** Histological distribution of the cases in this study.

| Histology | Counts (Percentage) |
|---|---|
| Meningothelial | 24 (51.1%) |
| Transitional | 14 (28.9%) |
| Fibrous | 4 (8.5%) |
| Psammomatous | 1 (2.1%) |
| Microcystic | 1 (2.1%) |
| Atypical | 3 (6.4%) |
| All | 47 (100%) |

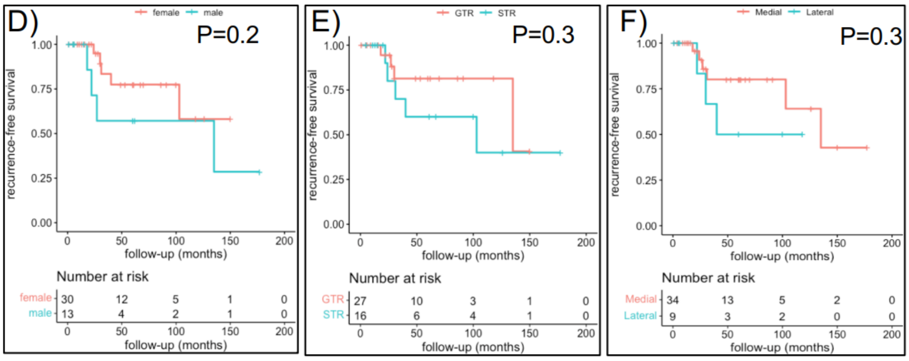


**Figure S1.** Kaplan-Meier plots of time to recurrence. Comparison between (**D**) gender, (**E**) EOR, and (**F**) location.
